# Effect of a Standard vs Enhanced Implementation Strategy to Improve Antibiotic Prescribing in Nursing Homes: A Trial Protocol of the Improving Management of Urinary Tract Infections in Nursing Institutions Through Facilitated Implementation (IMUNIFI) Study

**DOI:** 10.1001/jamanetworkopen.2019.9526

**Published:** 2019-09-11

**Authors:** James H. Ford, Lillian Vranas, DaRae Coughlin, Kathi M. Selle, Susan Nordman-Oliveira, Brenda Ryther, Tola Ewers, Victoria L. Griffin, Anna Eslinger, Joe Boero, Paula Hardgrove, Christopher J. Crnich

**Affiliations:** 1 School of Pharmacy, University of Wisconsin, Madison; 2 School of Medicine and Public Health, University of Wisconsin, Madison; 3 Center for Health Systems Research and Analysis, University of Wisconsin, Madison; 4 Wisconsin Department of Health Services, Division of Quality Assurance, Bureau of Education Services & Technology, Madison; 5 Marshfield Medical Center, Eau Claire, Wisconsin; 6 Wisconsin Healthcare-Associated Infections in Long-Term Care Coalition, Madison; 7 Aurora Health Care, West Allis, Wisconsin; 8 William S. Middleton Veterans Administration Hospital, Madison, Wisconsin

## Abstract

**Importance:**

Suspicion of urinary tract infection (UTI) is the major driver of overuse and misuse of antibiotics in nursing homes (NHs). Effects of interventions to improve the recognition and management of UTI in NHs have been mixed, potentially owing to differences in how interventions were implemented in different studies. An improved understanding of how implementation approach influences intervention adoption is needed to achieve wider dissemination of antibiotic stewardship interventions in NHs.

**Objective:**

To compare the effects of 2 implementation strategies on the adoption and effects of a quality improvement toolkit to enhance recognition and management of UTIs in NHs.

**Design, Setting, and Participants:**

This cluster-randomized hybrid type 2 effectiveness-implementation clinical trial will be performed over a 6-month baseline (January to June 2019) and 12-month postimplementation period (July 2019 to June 2020). A minimum of 20 Wisconsin NHs with 50 or more beds will be recruited and randomized in block sizes of 2 stratified by rurality (rural vs urban). All residents who are tested and/or treated for UTI in study NHs will be included in the analysis. All study NHs will implement a quality improvement toolkit focused on enhancing the recognition and management of UTIs. Facilities will be randomized to either a usual or enhanced implementation approach based on external facilitation (coaching), collaborative peer learning, and peer comparison feedback. Enhanced implementation is hypothesized to be associated with improvements in adoption of the quality improvement toolkit and clinical outcomes. Primary outcomes of the study will include number of (1) urine cultures per 1000 resident days and (2) antibiotic prescriptions for treatment of suspected UTI per 1000 resident-days. Secondary outcomes of the study will include appropriateness of UTI treatments, treatment length, use of fluoroquinolones, and resident transfers and mortality. A mixed-methods evaluation approach will be used to assess extent and determinants of adoption of the UTI quality improvement toolkit in study NHs.

**Discussion:**

Knowledge gained during this study could help inform future efforts to implement antibiotic stewardship and quality improvement interventions in NHs.

**Trial Registration:**

ClinicalTrials.gov identifier: NCT03520010

## Key Points

**Question:**

Does external facilitation improve adoption and effects of a complex antibiotic stewardship intervention in nursing homes?

**Findings:**

This trial protocol describes a cluster-randomized hybrid type 2 effectiveness-implementation clinical trial of implementation of a multicomponent toolkit focused on improving the recognition and management of suspected urinary tract infection (UTI) in nursing homes. The trial seeks to evaluate whether delivery of external facilitation—coaching, collaborative learning, and peer comparison feedback—to implement this toolkit results in higher rates of toolkit adoption and reduced rates of urine testing and initiation of antibiotics for treatment of suspected UTI.

**Meaning:**

If successful, external facilitation could become an effective approach for improving spread and adoption of antibiotic stewardship interventions, as well as other quality improvement initiatives, in the nursing home setting.

## Teaser

This trial protocol describes a randomized clinical trial intended to compare 2 strategies for implementing a toolkit for enhancing recognition and management of urinary tract infections in nursing homes.

## Introduction

Antibiotics are among the most commonly prescribed medications in nursing homes (NHs). More than half of individuals who reside in an NH for 6 months or longer will be prescribed at least 1 course of antibiotics.^1^ Suspicion of urinary tract infection (UTI) is the most common trigger for prescription of antibiotics in NHs and the condition most commonly associated with inappropriate antibiotic use in these facilities.^2^ Improving the recognition and management of UTI has, therefore, been identified as a major need in NHs.^3,4,5^

Recently, a stakeholder group comprising health care professionals from long-term care facilities as well as partners from academia and public health in Wisconsin^6^ have developed a quality improvement toolkit focused on enhancing staff and health care professional practices around recognition and management of UTI in NHs (hereafter, referred to as the *Wisconsin UTI Improvement Toolkit*). This toolkit is built around a previously published decision-support algorithm^2^ for managing suspected UTI and contains a number of tools and resources for improving the practice and interactions between nursing staff and health care professionals in situations in which UTI is suspected. Prior efforts to improve UTI recognition and management in NHs using decision support tools similar to those used in the Wisconsin UTI Improvement Toolkit have met with mixed results.^7,8,9,10,11,12^ The heterogeneous effects of behavioral interventions across different health care settings is not unique to NHs and likely reflects complexity in the interactions between the intervention, the context into which the intervention is being introduced, and the strategies used to facilitate its implementation.^13^ Despite improvements in specification of implementation strategies,^14,15^ knowledge about their mechanisms and effects on uptake of behavioral interventions remains rudimentary.^16^ The challenges of implementing behavioral interventions is particularly germane to NHs, which lack local access to individuals with expertise in quality improvement^17^ and face higher levels of staff turnover^18^ compared with other health care settings.

Studies focused on implementation of quality improvement interventions in NHs are rare.^19^ While several studies have identified factors that have contributed to lack of fidelity to UTI improvement interventions in NHs,^11,12,20^ we are unaware of any studies that have explicitly studied the effects of different implementation strategies on UTI improvement intervention uptake in NHs. External facilitation (also referred to as practice facilitation or coaching),^21^ peer learning collaboratives (also referred to as quality improvement collaboratives),^22^ and peer comparison feedback^23^ are 3 implementation strategies that have been shown to positively affect uptake of behavioral interventions in health care settings. The Improving Management of UTIs in Nursing Institutions Through Facilitated Implementation (IMUNIFI) study described in this protocol is designed to test the hypothesis that an enhanced implementation approach based on these 3 implementation supports will be associated with greater adoption of the Wisconsin UTI Improvement Toolkit and improve its impact on targeted behavioral outcomes compared with a standard implementation approach that does not include these additional supports.

## Methods

### Study Aims

The first aim of the study is to assess the effect of the Wisconsin UTI Improvement Toolkit on the recognition and management of UTI in a sample of Midwestern NHs. Measures of testing (urine culture order rate) and treatment (UTI treatment rate) behaviors, which are the primary targets of the intervention, will be used to assess toolkit effects.

The second aim of the study is to compare the effects of 2 different facilitation approaches on uptake and effectiveness of the Wisconsin UTI Improvement Toolkit. Study NHs will be randomly assigned to 1 of 2 facilitation approaches (usual vs enhanced), which are described further in this article. Primary outcome measures observed in each group will be compared to detect whether there are statistically significant differences between the 2 study implementation groups.

### Overall Study Design

**Figure 1.**
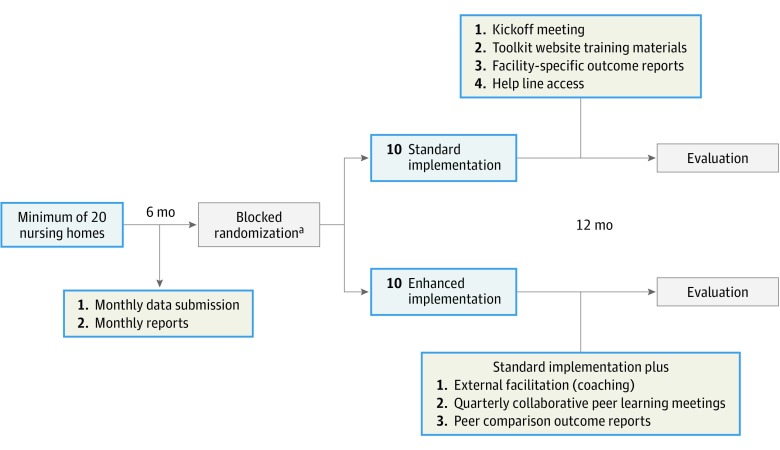
Study Overview ^a^Stratified by rurality in block sizes of 2.

A hybrid type 2 effectiveness-implementation cluster randomized trial of NHs that have elected to implement the Wisconsin UTI Improvement Toolkit (Figure 1) will be used in this study. This design is appropriate when the objective is to evaluate clinical effects of an intervention and the influence of context and/or facilitation strategies on intervention adoption.^24^ Following a 6-month baseline period, study NHs will be randomized to either a usual (control) or enhanced implementation (intervention) group based on external facilitation, peer learning sessions, and peer comparison feedback. Implementation rollout will occur simultaneously in all NHs. The study will compare urine testing and UTI treatment rates in the 2 study groups. The extent and determinants of adoption of the Wisconsin UTI Improvement Toolkit in study NHs will be assessed using a mixed-methods approach. The full trial protocol is available in Supplement 1.

### Ethical Considerations

The Human Subjects Institutional Review Board at the University of Wisconsin School of Medicine and Public Health approved this study protocol. Given the focus on quality improvement that targets health care practitioners, a waiver of informed consent was requested and approved.

### Participants

Nursing staff and practitioners in study NHs are the targets of the Wisconsin UTI Toolkit Improvement intervention. While not primary targets, all residents of NHs who develop a change-in-condition potentially caused by a UTI will potentially be affected by the intervention.

### Setting

At least 20 Wisconsin NHs, purposively selected for their geographic location across the state and to achieve a balance between rural and urban facilities, will be recruited. The rationale for this sampling approach is that rural NHs are less likely to be members of an NH network and may have limited access to improvement resources and expertise. This approach will enhance our capacity to identify barriers to implementation and further disseminate the toolkit. Facilities will be recruited through a listserv maintained by the Wisconsin Department of Health Services and through referrals from members of for-profit and not-for-profit NH advocacy associations in Wisconsin.

### Inclusion Criteria

Medicare- and Medicaid-certified NHs with 50 or more long-term care and skilled nursing beds in Wisconsin will be eligible for this study. The NHs that agree to participate will be asked to submit monthly data on facility urine cultures and UTI treatment events through a web-based portal designed for this study during a baseline period before implementing the Wisconsin UTI Improvement Toolkit. Facilities that demonstrate commitment by submitting 3 sequential months of preintervention data will remain eligible for randomization.

### Exclusion Criteria

Facilities less than 50 beds in size, those that primarily provide advanced specialty care (ventilator or strict rehabilitation care), and assisted living facilities will be excluded. Nursing homes that otherwise meet inclusion criteria but fail to submit 3 sequential months of baseline data will not be randomized.

### Study Intervention

The Wisconsin UTI Improvement Toolkit is a multicomponent suite of tools, resources, and best practices structured around a previously published decision support algorithm for managing suspected UTI.^2^ The behavioral objectives of the decision support algorithm used in the toolkit are similar to those used in other UTI improvement interventions previously tested in the NH setting.^7,8,9,10,11,12^

**Figure 2.**
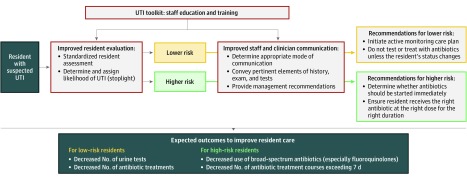
Wisconsin UTI Improvement Toolkit Logic Model UTI indicates urinary tract infection.

The components of the toolkit focus on enhancing the development of professional human resources in NHs and target both nursing staff and clinicians. The tools and resources that compose the Wisconsin UTI Improvement Toolkit are arranged in modules that focus on different aspects of UTI recognition and management (Table 1). The behavioral objectives of the toolkit are to (1) improve nursing staff assessment of residents experiencing a change in condition potentially attributable to UTI; (2) improve the quality of interprofessional communication when a diagnosis of UTI is being considered; (3) ensure that nursing staff and clinicians ascertain the likelihood of UTI (low vs high probability); (4) defer urine testing and antibiotic therapy when the probability of UTI is low; and (5) prescribe antibiotics appropriately (choice of agent and treatment duration) when the probability of UTI is high (Figure 2).

**Table 1.** Structure of the Wisconsin UTI Improvement Toolkit

Module Name and Sections	Objectives	Tools
**Module 1. Overview and Rationale**		
Overview Clinical rationale Regulatory rationale	Provide an overview of the toolkit Explain why antibiotic stewardship matters from clinical and regulatory perspectives Educate clinicians, nursing staff, and family members of residents on appropriate management of UTIs	Slide sets and videos Staff and clinician brochure Family education brochure Family education letter and video
**Module 2. How to Prevent Catheter-Associated UTI**		
Background and risk factors Appropriate indications for indwelling catheter use Indwelling catheter insertion and maintenance	Provide guidance on appropriate use and management of indwelling urinary catheters Provide guidance on how to properly collect a urine specimen from a resident with a urinary catheter	Slide sets and videos Resources on catheter insertion Resources on catheter maintenance Resources on hand hygiene
**Module 3. When to Test a Urine Specimen**		
What is a UTI? When to submit a urine specimen for testing Case studies Suggested educational plan	Provide guidance on how to reliably stratify residents into low and high risk of UTI Increase nursing staff comfort with communicating assessment findings to health care professionals and making recommendations for actions based on UTI risk Provide guidance on how to perform active monitoring for residents with a low risk of UTI	Slide sets and videos When to test nursing tool Urine testing tracking sheet Scripts for contacting health care professionals Case studies Resources to support active monitoring procedures
**Module 4: When and How to Treat a UTI**		
When to treat? How to treat? How to modify?	Provide the rationale and benefits of active monitoring Provide guidance on antibiotic selection, dosage, and duration for treating a UTI Provide rationale and guidance for performing an antibiotic time-out after prescribing	Slide sets and videos How to treat provider tool Antibiotic time-out tool
**Module 5. Organizational Tools**		
Overview of quality improvement and how to lead change in the organization The importance of tracking and reporting data for organizational quality improvement Sustainability of organizational change	Provide guidance for assembling an improvement team Provide examples of tools for use in the long-term care facility to help change frontline staff and clinician behavior Explain how data tracking and reporting can be used for organizational quality improvement Discuss sustainability and the importance of developing a plan to sustain improvements	Sample policy for collaborative practice agreement Sample table for collaborative practice agreement Collection of urine specimens and urine test tracking spreadsheet

Abbreviation: UTI, urinary tract infection.

### Study Implementation Groups

#### Usual Implementation

Study NHs randomized to the usual implementation (control) will participate in a kickoff meeting. The meeting will introduce the different toolkit components and address concepts germane to implementation and their relationship to the NH Quality Assessment Process Improvement (QAPI) process.^25^ These NHs will have ongoing access to online training resources and improvement tools, including a web-based tool for tracking and visualizing facility urine culture and UTI treatment rates. Study NHs assigned to the usual implementation group will be able to contact the research team with questions but will not receive additional support (Table 2).

**Table 2.** Urinary Tract Infection Implementation Approach by Facilities

Implementation Strategy	Implementation Strategy Description	Control	Intervention
IMUNIFI website	Online portal to general information about the IMUNIFI project and frequently asked questions about the toolkit, data entry, and feedback reports	✓	✓
Interactive UTI toolkit	Online access to the 5 modules of the UTI toolkit	✓	✓
Interactive data entry and feedback reports	Online data entry of suspected UTI line list	✓	✓
	Individual nursing home feedback reports to track internal trends; report categories include urine cultures, antibiotic prescriptions, treatment duration, spectrum changes, and antibiograms		
Ask an expert	Ability to seek ad hoc advice via email about the UTI toolkit	✓	✓
In-person meeting: overview	Overview of how to use the UTI toolkit	✓	✓
	Overview of how to use and interpret facility data		
Enhanced feedback reports	Benchmark comparison reports related to urine cultures, antibiotic prescriptions, treatment duration, spectrum changes, and antibiograms		✓
In-person meeting: coaching	Overview of coaching expectations including explaining role of the coach and future interactions with the champion		✓
	Review study timeline		
	Overview of how to use and interpret facility benchmark data		
	Review the specifics of the coaching call—share template items		
Coaching	Nursing homes will participate in regular coaching calls that will review data reports, process components, and facilitators and barriers to adoption of the process and develop and ongoing action plan		✓
Collaborative learning	Quarterly webinars that will focus on interorganizational sharing of effective practices, discuss implementation barriers, and provide an opportunity to introduce new concepts or reinforce existing concepts		✓

Abbreviations: IMUNIFI, Improving Management of Urinary Tract Infections in Nursing Institutions Through Facilitated Implementation Study; UTI, urinary tract infection.

#### Enhanced Implementation

Study NHs randomized to the enhanced implementation (intervention) group will attend the same kickoff meeting and have access to the same online resources, but will also (1) be assigned a clinical coach, (2) have the opportunity to participate in peer learning activities, and (3) receive peer comparison feedback reports. The coaching support will be delivered by a trained nurse through regular telephone calls with a study NH staff member responsible for local implementation of the UTI toolkit (referred to as an internal champion). Initial calls will focus on reviewing current UTI diagnosis- and management-related workflows and identification of opportunities to integrate different aspects of the Wisconsin UTI Improvement Toolkit. Subsequent calls will focus on reinforcing important aspects of the QAPI process, including (1) establishing a change team; (2) identifying barriers and facilitators to change; (3) reviewing and interpreting primary outcomes feedback reports; (4) identifying and prioritizing future change efforts; and (5) developing a plan to sustain improvement efforts. The peer learning support will be delivered through quarterly webinars in which NH champions will be encouraged to discuss successes and challenges and share strategies for implementing the toolkit. The peer comparison feedback support will be delivered through the web-based data tracking and visualization tool described. Nursing homes assigned to the enhanced implementation group will be able to visualize their performance over time and see how they are performing relative to other participating NHs (Table 2).

### Study Outcomes 

#### Primary Outcomes

Urine culture orders per 1000 resident-days and antibiotic prescriptions for treatment of suspected UTI per 1000 resident-days will be the co–primary outcomes assessed. Urine cultures triggered by an abnormal urinalysis result, even if obtained for reasons other than suspicion of UTI, will be included as an outcome. The treatment indication included in clinician orders and documentation will be used to define antibiotic prescriptions for treatment of suspected UTI (Table 3).

**Table 3.** Study Outcomes and Control Variables

Domains	Measures	Data Sources
Outcomes		
Primary clinical	Urine cultures per 1000 resident-days	Facility self-report via data submission portal
	Antibiotic prescriptions for UTI per 1000 resident-days	
Secondary clinical	Days of therapy for UTI per 1000 resident-days	Facility consultant pharmacies
	Fluoroquinolone antibiotic prescriptions and days of therapy per 1000 resident-days	
	% of urine cultures meeting appropriateness criteria	Facility self-report via data submission portal
	% of antibiotic prescriptions for UTI meeting appropriateness criteria	
	% Urine cultures positive for resistant bacteria	Facility reference laboratories
	Number of positive *Clostridium difficile* tests	
	Hospital or emergency department transfers per 1000 resident-days	Facility self-report
	Resident deaths per 1000 resident-days	
Implementation	Facility staff participation in meeting and coaching calls	Attendance and coaching call logs
	Consistency of clinical data submission	Weblogs
	Use of the toolkit website	
	Intensity of staff and clinician education	Survey instrument administered to frontline staff
	Use of intervention tools	
	Knowledge, attitudes, and perceptions about the toolkit	
	Implementation barriers and facilitators	Stakeholder interviews and coaching call logs
	Challenges to implementation and strategies to overcome them	
Facility demographic characteristics	Facility nursing staff team climate and communication	Survey instrument administered to frontline staff
	Leadership turnover	Survey instrument administered to facility leadership staff
	Existing antibiotic stewardship and infection prevention activities	
	Bed size, ownership status, skilled nursing services provided, resident complexity, and Medicare star ratings	State and national administrative data records
	Staff turnover and retention rates	State of Wisconsin Consumer Information Reports

Abbreviation: UTI, urinary tract infection.

#### Secondary Outcomes

The secondary clinical outcomes will include (1) days of therapy for treatment of suspected UTI per 1000 resident-days; (2) fluoroquinolone antibiotic prescriptions and days of therapy per 1000 resident-days for treatment of suspected UTI; (3) percentage of urine cultures meeting Wisconsin UTI Improvement Toolkit criteria; (4) percentage of antibiotic prescriptions for UTI meeting the Wisconsin UTI Improvement Toolkit criteria^2,26^; (5) percentage of urine cultures positive for bacteria resistant to common first-line and second-line oral antibiotics; (6) *Clostridioides difficile–*positive tests per 1000 resident-days; (7) transfers to hospitals or emergency departments per 1000 resident-days; and (8) resident deaths per 1000 resident-days.

Measures of adoption of the Wisconsin UTI Improvement Toolkit in study NHs will include (1) staff participation in study meetings and coaching calls; (2) consistency of clinical data submission; (3) toolkit website use; (4) intensity of staff and clinician education; (5) use of intervention tools; and (6) knowledge, attitudes, and perceptions about the toolkit. Potential determinants of adoption of the Wisconsin UTI Improvement Toolkit, including barriers and facilitators, will be elicited from nursing staff, clinicians, and facility leadership (referred to as key informants) (Table 3).

### Data Collection Methods

A mixed-methods approach will be used to collect data in this study. Information sources will include (1) data submitted through the web-based data portal; (2) surveys administered to internal champions, nursing staff, and clinicians; (3) aggregated data obtained from pertinent administrative, laboratory, and pharmacy records; and (4) interviews and focus groups with key informants in study NHs. Table 3 provides a summary of study outcomes and the data sources used to derive these outcomes. A detailed description of the project key tasks and timeline is given in the eTable in Supplement 2.

#### Facility Demographic Characteristics

Each study NH will complete a facility-level survey that captures information on characteristics of leadership as well as structure and process of existing antibiotic stewardship and infection prevention efforts. Additional information on geographic location, bed size, ownership status, skilled services provided (eg, parenteral therapy and wound care), resident complexity,^27^ staffing turnover and retention measures, and Medicare star rating^28^ will be abstracted from existing state and national administrative data records. Frontline nursing staff in study NHs will be asked to complete a survey on existing team climate and aspects of communication around identification and reporting of changes in condition of residents that may require antibiotic therapy. This survey will be administered at the time of randomization as well as 6 and 12 months following implementation of the Wisconsin UTI Improvement Toolkit.

#### Clinical Outcomes

The internal champion from each study NH will submit data on the number of resident-days, urine cultures, antibiotic prescriptions, and clinical information to ascertain if these meet Wisconsin appropriateness criteria via a web-based data submission portal on a monthly basis. Champions will be trained to use the data submission portal via a webinar. The portal will include frequently asked questions and help desk access for technical issues related to data submission. Each NH will be asked to submit test records, and members of the research team will follow up with NHs to identify problems with data submission prior to randomization.

The research team will obtain aggregate data on antibiotic prescriptions from the consultant pharmacist to determine days of antibiotic therapy ordered per 1000 resident-days as well as the fluoroquinolone prescriptions and days of fluoroquinolone antibiotic therapy ordered. Deidentified aggregate data on urine culture results from laboratories used by study NHs will be obtained to cross-validate self-reported rates of urine culture orders and characterize antibiotic resistance patterns. Results for *C difficile* tests at a facility level will be obtained in a similar manner. The research team will work with facility administrative staff to collect data on monthly rates of resident transfers and mortality.

#### Measures and Determinants of Toolkit Adoption

Webinar and meeting attendance as well as coaching call records will be used to measure study NH participation in group and coaching call activities. Weblogs will be used to measure consistency of study NH data submission and frequency with which staff access the different training modules within the UTI toolkit and review their feedback reports as well as other components of the IMUNIFI website.

A survey instrument will be used to assess (1) intensity of staff education activities; (2) reported use of different toolkit resources (informational posters, family and practitioner educational materials, and decision aids); (3) staff perceptions about the usefulness and effects of the Wisconsin UTI Improvement Toolkit; and (4) staff perceptions about the likelihood of sustaining the intervention. The survey instrument will be administered to frontline nursing staff (registered nurses and licensed practical nurses) via email during months 6 and 12 of the postimplementation period. A second email followed by telephone calls to facility leadership will be used to enhance survey response. Based on facility staffing levels and a projected response rate of 50%, we anticipate that 160 surveys will be completed at each point.

Interviews with key informants in 3 to 4 high- and low-performing study NHs, determined by rank order of the primary study outcomes, will be performed to identify specific barriers and facilitators to implementation of the Wisconsin UTI Improvement Toolkit. Information from logs created during coaching calls will be used to identify common challenges to implementation encountered in NHs assigned to the enhanced implementation arm of the study and the types and effectiveness of strategies that the internal champion and coaches had codeveloped to overcome these challenges.

### Statistical Analysis

#### Sample Size

Based on data previously collected by our group,^29^ the expected rate of UTI treatment is approximately 4.0 per 1000 resident-days. Assuming 10 facilities of equal cluster sizes in each study group and an intraclass correlation (ρ) of 0.49, we estimate our study will have 90% power to detect a 15% reduction in the UTI treatment rate (antibiotic prescriptions for UTI per 1000 resident-days). We plan to recruit at least 35 NHs and randomize at least 26 NHs to account for a 25% facility dropout rate before and after randomization.

#### Randomization

Randomization will be computer generated and stratified based on geographic location (urban vs rural) in block sizes of 2. An individual uninvolved with the study will be responsible for generating the randomization and allocation of NHs to the 2 treatment groups of the study. Given the nature of the intervention, neither the participants nor the investigators will be blinded during this study.

#### Analyses of Clinical Outcomes

Generalized estimating equations using segmented regression and a Poisson distribution accounting for autocorrelation of data within facilities will be used to evaluate the 2 primary outcomes across all study sites in aggregate. Four estimates from these models will be evaluated: (1) the preintervention trend (β_1_); (2) the immediate change in the outcomes following the kickoff webinar (β_2_); (3) the difference between preimplementation and postimplementation trend (β_3_); and (4) the postimplementation outcome rate change (β_1_ + β_3_). First- and second-order autocorrelation will be assessed using the Durbin-Watson statistic. Robust standard errors will be used to estimate variance. Similar generalized linear models will be fit to analyze secondary and safety outcomes. To assess the effect of enhanced implementation on the effectiveness of the toolkit, the segmented regression model developed will be extended to include a covariate for the study implementation group (usual implementation = 0; enhanced implementation = 1). We will explore adding control variables into our models if significant imbalances in NH characteristics (eg, size, staffing ratios, profit status, geographic location, Centers for Medicare & Medicaid Services 5-star rating) and level of intervention adoption (described in the following section) are identified in our preliminary analyses.

#### Analyses of Toolkit Adoption Measures and Determinants

Data from study event attendance records, weblogs of toolkit website use, frontline staff responses to survey questions focused on familiarity, and use of the different toolkit elements will be integrated into a measure that stratifies randomized NHs into 3 levels of intervention adoption (high, medium, and low). The generalized estimating equation models described above will be extended by including the facility adoption measure as an independent variable to assess the relationships between toolkit adoption and the primary and secondary study outcomes.

Determinants of toolkit adoption will be identified through qualitative analyses of key informant interviews and coach notes. Interviews with key informants in high- and low-performing NHs will be recorded, deidentified, and transcribed verbatim. A mixed deductive and inductive thematic analysis^30^ guided by the Systems Engineering Initiative for Patient Safety framework^31^ will be used to analyze interview data. The Systems Engineering Initiative for Patient Safety framework is a human factors engineering framework that has been used extensively in a variety of health care settings to characterize work systems and document changes to the work system following implementation of quality improvement interventions.^32,33,34,35^ Interview transcripts will be coded independently with tests for reliability. Analyses will focus on identifying the changes (or lack thereof) that occurred in study NH work systems as a result of implementing the Wisconsin UTI Toolkit in an effort to identify key barriers and facilitators to adoption. A similar approach will be used in the analyses of coach notes to assess the effects of coaching on toolkit adoption. We will use the comparative conceptual framework proposed by Bond and Seneque,^36^ which suggests that organizational coaching occurs across 1 of 5 domains (managerial, consulting, mentoring, facilitating, or coaching), to classify the advice provided during the coaching interactions. Members of the research team will attempt to integrate information from the different data sources using a triangulation approach^37^ to better explain the relationships between the 2 implementation approaches, adoption of the Wisconsin UTI Improvement Toolkit, and observed effects on clinical outcomes.

## Discussion

During this project, we expect to demonstrate significant reductions in antibiotic use for treatment of suspected UTI in study NHs following adoption of the Wisconsin UTI Improvement Toolkit. We also expect that NHs that receive externally facilitated support during the implementation of the toolkit will demonstrate greater levels of adoption as well as greater reductions in urine culture testing and antibiotic use compared with NHs that receive usual support during implementation. We further expect to generate important information on the types of barriers NHs encountered during the implementation and the strategies they found to be most successful for overcoming these barriers. Finally, we expect to generate knowledge on the mechanisms by which enhanced implementation influenced adoption of the Wisconsin UTI Improvement Toolkit and, in turn, the influence of adoption on clinical outcomes in study NHs.

At project conclusion, we will have (1) assessed the effects of enhanced implementation on adoption of evidence-based antibiotic stewardship practices in Wisconsin NHs and (2) determined the key facilitators and barriers associated with the 2 implementation approaches used. The knowledge generated from this project could benefit subsequent projects focused on dissemination of the Wisconsin UTI Improvement Toolkit to other facilities in the state and facilitating implementation of other types of interventions to improve the quality of antibiotic prescribing in NHs. The intervention implemented in this study is complex and our findings may not be applicable to the implementation of simple interventions. However, many of the problems encountered in NHs are complex in nature, including other medication safety problems (eg, antipsychotic medications), care transitions, and fall prevention. Consequently, we believe this study will generate knowledge that can support the implementation of interventions to address these, as well as other, important problems.

## Notes

Supplement 1. **Trial Protocol**

Supplement 2. **eTable. **Project Timeline and Key Tasks
