# Supplementary material for: Effect of a Standard vs Enhanced Implementation Strategy to Improve Antibiotic Prescribing in Nursing Homes: A Trial Protocol of the Improving Management of Urinary Tract Infections in Nursing Institutions Through Facilitated Implementation (IMUNIFI) Study
Source: JAMA Netw Open. 2019 Sep 11;2(9):e199526. doi: 10.1001/jamanetworkopen.2019.9526 (PMC6739723; doi:10.1001/jamanetworkopen.2019.9526)
Supplement: Supplement 1. — Trial Protocol [file jamanetwopen-2-e199526-s001.pdf]

A Cluster Randomized Trial to Assess the Impact of Facilitated Implementation on Antibiotic Stewardship in Wisconsin Nursing Homes

Primary Investigator: Christopher J. Crnich, MD PhD
Chief of Medicine, Madison VA Hospital
Associate Professor, Department of Medicine
University of Wisconsin- Madison

Co-Investigator: Jay Ford, PhD
Assistant Professor, School of Pharmacy
University of Wisconson-Madison

Study Protocol

I. Background

Antibiotic resistance is one of the three greatest threats to human health according to the World Health Organization [1]. Antibiotic-resistant bacteria and *Clostridium difficile* are responsible for 2.3 million infections and 37,000 deaths in the U.S. every year [2]. Antibiotics are one of the most commonly prescribed medications in nursing homes (NHs). Up to 40% of NH residents are colonized with antibiotic-resistant bacteria [3], and NHs play a critical role in the emergence and spread of antibiotic resistance and *C. difficile* in the communities they serve [4-6]. Antibiotics are prescribed to 65% of individuals who reside in a NH for ≥ 6 months [7]. Treatment of suspected urinary tract infection (UTI) accounts for nearly half of the antibiotic use in Wisconsin NHs [1]. However, there is compelling evidence that antibiotic prescribing patterns in NHs is sub-optimal (5). Less than half of the antibiotics prescribed for UTI in Wisconsin NHs are justified [6], and improving the management of UTI in NHs has been identified as a major priority by experts in the field [7-9].

We along with our community partners have developed a toolkit of evidence-based practices for improving the management of UTIs in Wisconsin NHs (hereafter referred to as the Wisconsin UTI Improvement Toolkit). While use of the practices incorporated in the Wisconsin UTI Improvement Toolkit has resulted in notable improvements in the quality of antibiotic prescribing for the treatment of UTI in a number of studies [10-12], more limited effects have been observed in other studies despite employing similar improvement strategies [13-15]. Heterogeneity of effects across different settings is a problem commonly encountered with healthcare behavioral interventions and may be due to characteristics of the intervention, the setting into which it is being introduced, and the method of implementing the intervention in that setting [16]. There are a number of challenges to improving quality in NHs, including lack of improvement expertise [17] and high levels of staff turnover [18] to name a few. It is unrealistic to expect that these structural barriers will abate anytime soon. Consequently, it is critically important to identify effective strategies to overcome them if meaningful improvements in the quality of antibiotic prescribing in NHs are to be achieved.

II. Study Objectives

<u>Our long-term objective</u> is to improve the quality and safety of antibiotic prescribing in Wisconsin NHs. The objective of the current project, which is a critical step in the pursuit of our long-term objective, is to identify effective strategies for supporting the implementation of antibiotic stewardship interventions in NHs. We hypothesize that externally-facilitated implementation based on coaching and peer-to-peer learning will be associated with: (**hypothesis 1**) better adoption of the Wisconsin UTI Improvement Toolkit and (**hypothesis 2**) reduced rates of inappropriate antibiotic use as compared to a usual implementation approach that relies predominantly on internal improvement resources available in NHs. We base these hypotheses on studies demonstrating more effective uptake of evidence-based practices with coaching [19-22] and peer-to-peer learning [23-25] in other healthcare settings, particularly resource-limited settings. A preliminary analysis of qualitative data collected from another of the PI's research projects (Agency for Healthcare Research and Quality 1R18 HS022465-01) indicates that clinical coaching is helping champions in study NHs identify barriers to change and develop more effective strategies for overcoming these barriers.

The primary study objectives are to assess the overall impact of the UTI Improvement Toolkit on antibiotic utilization and determine the extent to which effects vary by the implementation approach employed. We will achieve these two objectives through the following specific aims:

Specific Aim 1: Evaluate the impact of the Wisconsin UTI Improvement Toolkit on antibiotic prescribing for UTI in Wisconsin NHs.

Specific Aim 2: Assess the influence of external facilitation on the adoption and the effects of the Wisconsin UTI Improvement Toolkit in Wisconsin NHs.

At project conclusion, we will have (1) assessed the effects of externally-facilitated implementation on the adoption of evidence-based antibiotic stewardship practices in Wisconsin NHs, and (2) determined the key facilitators and barriers associated with the two implementation approaches employed.

III. Study Methods

A. Overall Study Design and plan

We will introduce the Wisconsin UTI Improvement Toolkit (see Section III.B. Wisconsin UTI Improvement Toolkit) in 20 Wisconsin NHs and assess its impact on antibiotic utilization using segmented regression. Two different implementation strategies (see Section III.C. Study Arms) will be employed during the introduction of the Wisconsin UTI Improvement Toolkit in study NHs using a hybrid type 2 effectiveness-implementation study design in which half of the NHs will be randomized to usual (internally-driven) implementation with the other half randomized to externally-facilitated implementation based on coaching and peer-to-peer learning. The effects of the two implementation approaches will be assessed using a mixed methods approach.

B. Wisconsin UTI Improvement Toolkit

The PI (Crnich) has worked with academic, community and public partners in Wisconsin to develop a multi-component toolkit to improve the management of UTIs in NHs. The WI Healthcare Associated Infections (HAI) in Long term care (LTC) UTI toolkit was developed through a rigorous review of published theory and evidence [7] with input from frontline clinicians who practice in Wisconsin NHs and is structured around a UTI management framework developed by the PI [31]. The toolkit targets three major nursing staff and provider behaviors: (a) standardization of the assessment of the resident experiencing a change-in-condition; (b) implementation of delayed testing and treatment of residents experiencing a low-risk change-in-condition; and (c) appropriate selection of antibiotics (drug, dose, duration) in those residents where the likelihood of UTI is high. Education, communication, and decision-support tools have been developed in order to achieve improvements in each of these behavioral domains, and the toolkit also includes recommended practices to help prevent UTIs as well as process and outcome tracking tools designed to help staff in NHs assess implementation progress. Further details about the UTI toolkit are provided in the Appendix.

C. Study Arms

C.1. Control Arm: Internally-driven implementation

Study NHs randomized to the control arm will participate in kickoff meetings introducing the different UTI toolkit components. The meeting will also cover topics on how to assemble a change team, how to use the different tools in the UTI toolkit, and how to track process and outcome measures to monitor progress. NHs assigned to the control arm will have access to a web-based data tracking tool that will allow them to trend a number of study outcomes (e.g., number of monthly urine cultures and UTI treatment events) in their facility over time. Instructions and educational tools will be made available as an online resource but otherwise NHs in the control arm will be expected to implement the Wisconsin UTI Improvement Toolkit using existing internal resources.

C.2. Intervention Arm: Externally-facilitated implementation

NHs randomized to the externally-facilitated arm will attend the same kickoff meetings and have access to the same online resources as the control arm but will also be assigned a clinical coach and will have the opportunity to participate in a series of hosted peer-to-peer learning opportunities. The clinical coach will meet regularly with NH staff to guide the facility through implementation of the Wisconsin UTI Improvement Toolkit. Coach supports will occur during a site visit and individual coaching calls.

C.2.1. Site Visit

At the site visit, the coach will review the current UTI diagnosis and management-related workflows and work with SNF staff to identify opportunities to integrate different aspects of the Wisconsin UTI Improvement Toolkit into these existing workflows.

C.2.2. Individual Coaching Calls

Following the site visits, the coach will conduct bi-weekly phone calls with staff in study NHs. These calls will focus on a number of topics, including:

a) Establishing a change team;
b) Identification of barriers and facilitators of change;
c) Implementation of changes to support the ongoing integration of the toolkit practices into facility workflows;
d) Review and interpretation of primary outcomes feedback reports;
e) Identification and prioritization of future change efforts;
f) Development of a plan to sustain improvement efforts following completion of the study.

C.2.3. Peer-to-Peer Learning Meetings

The research team will host a series of learning meetings during which champions in intervention NHs will be encouraged to discuss successes/challenges and share ideas and strategies they employed to implement the toolkit. Participants will also be encouraged to collaborate with their peers in other facilities outside the formal peer-to-peer meetings.

C.2.4. Enhanced Feedback Reports

Intervention NHs will be able to track their data using the web-based tracking tool that will be accessible by the control NHs. However, the feedback reports made available to intervention NHs will include information on the performance of other participating NHs (in aggregate) allowing the intervention NHs to trend their own data and benchmark themselves against their peers.

D. Study Sites

D.1. Overview

20 Wisconsin NHs, purposively selected for their geographic location to achieve a balance between rural and urban facilities, will be selected for participation in this study. Our rationale for employing this sampling approach is that rural NHs are less likely to be members of a NH network and may have more limited access to improvement resources and expertise. This approach will enhance our capacity to identify barriers to implementation that will be encountered in NHs throughout Wisconsin and enhance future toolkit dissemination efforts.

D.1.1. Inclusion criteria

a) Medicare and Medicaid (dually) certified nursing homes
b) Long-term care and skilled nursing beds > 50 beds
c) The management of the facility agrees to random allocation to control or intervention group
d) The facility is able to submit 3 sequential months of data on facility urine culture and antibiotic treatment rates via the study web portal

D.1.2. Exclusion criteria

a) Assisted living facility wards
b) Specialty care (ventilator or strict rehabilitation) wards

D.2. Recruitment

Facilities have been recruited through referrals from members of the Wisconsin Healthcare-Associated Infections in Long-Term Coalition as well as Wisconsin nursing home associations (Leading Age and Wisconsin Health Care Association).

D.3. Randomization

Randomization will be computer generated and stratified based on geographic location (urban vs. rural) in block sizes of two.

E. Study Outcomes

E.1. Overview

While the individual components of the Wisconsin UTI Improvement Toolkit are evidence-based, their combined effects on antibiotic prescribing are not known. We will assess the combined effects of the toolkit by comparing UTI treatment rates in study NHs before and after implementation of the toolkit using segmented regression methods. A number of data measures will be employed during this study.

Focus Area	Domain	Measure(s)	Data Source
Nursing Home Level Measures	Facility demographics	Facility demographics (location, size, services, etc.)	UW NH Org Profile
		Resident characteristics (%post-acute, payer, etc.)	CASPER database
		Nursing staffing levels (CNA, LPN, RN)	
		Nursing retention measures	
	Work system	People, tools, tasks, environment	Interviews & observation
		Process and information flow maps	
		ASP Structure and Process	UW ASP Survey
	Organizational culture	Readiness for change	UW organizational survey
		Management support of frontline staff	
		Perceived capacity to sustain change	
NH Level Measure	Reach	# of NHs adopting the UTI toolkit with fidelity	Interviews and surveys
		AS & DOT for UTI per 1,000 resident-days	Facility supplied

Resident Level Outcomes	Antibiotic measures (Effectiveness)	FQ-AS and FQ-DOT per 1,000 resident-days	
		% of AS for UTI meeting appropriateness criteria	
		% urine cultures positive for resistant bacteria	
		Number of positive Clostridium difficile tests	
	Testing measures (Effectiveness)	Urine cultures per 1,000 resident-days	Facility supplied
		% of urine cultures meeting appropriateness criteria	
	Safety measures	Hospital/ED transfers per 1,000 resident-days	Facility supplied
		Resident deaths per 1,000 resident-days	
Nursing Home Quality Indicators	Quality indicators (Effectiveness)	Residents with UTI; Prevalence of UTI infection (only) and all infections	CHSRA/DHS Nursing Home Scorecard
	F-Tag Citations (Effectiveness)	F-Tag 441 (Infection Control) and F-Tag 329 (Drug Regimen) as well as severity	CASPER
Staff Feedback	Adoption	Knowledge, attitudes, & perceptions about the toolkit<br>Participant feedback on implementation.	Interviews and surveys
NH and Staff Feedback	Implementation	UTI toolkit fidelity checklist<br>Implementation Barriers and Facilitators	Interviews and surveys
Maintenance	Resident and quality indicators	Sustainability facilitators/barriers<br>Short-term sustainment of clinical indicator improvements	Interviews and Effectiveness Outcomes

E.2. Study Measures

E.2.1. Primary Measures

a) Urine culture orders per 1,000 resident-days.
b) Antibiotic starts for treatment of suspected UTI per 1,000 resident-days.

E.2.2. Secondary Measures

a) Days of therapy for treatment of suspected UTI per 1,000 resident-days.
b) Fluoroquinolone antibiotic starts and days of therapy per 1000 resident-days.
c) Percentage of antibiotic starts for UTI meeting Wisconsin appropriateness criteria.
d) Percentage of urine cultures meeting Wisconsin appropriateness criteria.
e) Percentage of urine cultures positive for resistant bacteria.
f) Number of positive *clostridium difficile* tests.
g) Number of transfers to hospitals or emergency departments per 1000 resident-days.
h) Number of resident deaths per 1000 resident-days.

E.2.3. Facility Demographics

A variety of facility-level demographic data will be collected in order to assess comparability between control and intervention facilities. These will include:

a) Geographic location (rural versus urban)
b) Size (beds)
c) Skilled nursing services provided (e.g., parenteral therapy, wound care, etc)
d) Ratio of skilled to domiciliary care beds
e) Resident complexity (Resource utilization group [RUG])
f) Staffing ratios (registered nurse [RN], licensed practical nurse [LPN], certified nursing assistant [CNA])
g) Staff turnover (RN, LPN, CNA)
h) Existing facility antibiotic stewardship structure and process
i) Organizational culture
j) Nursing home quality measures

E.3. Data Collection Procedures

E.3.1. Primary Outcome Measures

Participating NHs will submit data on the number of urine cultures and antibiotic starts via a web-based portal on a monthly basis.

E.3.2. Secondary Outcome Measures

Participating NHs will submit data on the number of urine cultures and antibiotic starts meeting Wisconsin appropriateness criteria [cite] via a web-based portal on a monthly basis. The Wisconsin appropriateness criteria [cite] require residents to either exhibit symptoms that localize to the urinary tract (e.g., dysuria, frequency) or systemic signs of infection (e.g., fever, hypotension, etc.) in the absence of another obvious source of infection.

The research team will obtain aggregate data on antibiotic prescriptions from the facility consultant pharmacy in order to determine days of antibiotic therapy per 1,000 resident-days as well as the number of fluoroquinolone starts and days of fluoroquinolone antibiotic therapy.

The research team will obtain aggregate data on urine culture results to determine the rates of urine culture resistance in study nursing homes. Clostridium difficile results at a facility level will be obtained in a similar manner.

Facilities will be asked to provider their aggregate resident transfer and mortality data on a monthly basis.

E.3.3. Facility Demographics

A facility demographics survey will be employed to collect information on study NH size, staffing levels, and types of skilled nursing services provided, etc. The Certification and Survey Provider Enhanced Reporting (CASPER) database, a Center for Medicaid and Medicare Services database that provides information about nursing home demographics and selected quality indicators, will be accessed to determine study nursing home staff turnover, aggregate measures of resident complexity and facility F-tag deficiencies (F-tag 329 [inappropriate medication use] and 441 [infection control]). A survey instrument developed by the research team [cite] will be used to characterize each facility’s existing antibiotic stewardship structure and process. Another survey instrument developed by the research team [cite] will be employed to assess each facility’s organizational culture. We will collect data on the number and the percentage of residents with UTI, as well as on the prevalence of UTI infections and other infections using

the CHSRA/DHS Nursing Home Scorecard. This scorecard, which is being developed by the UW Center for Health Systems Research and Analysis (CHSRA), provides trends across a wide array of resident level outcomes for Wisconsin Nursing Homes. It also includes comparisons to peer and NHs across the state. The scorecard will be used to report changes in these outcomes.

E.4. Data Analyses

Generalized estimating equations using segmented regression and a Poisson distribution accounting for clustering by facility will be employed to evaluate the two primary outcomes across all study sites in aggregate. Four estimates from these models will be evaluated: 1) the pre-intervention trend (β_1); 2) the immediate change in the outcomes following the kickoff webinar (β_2); 3) the difference between pre- and post-implementation trend (β_3); and 4) the post-implementation outcome rate change ($\beta_1 + \beta_3$). First and second order autocorrelation will be assessed using the Durbin-Watson statistic. Robust standard errors will be used to estimate variance. Similar generalized linear models will be fit to analyze secondary and safety outcomes. To assess the impact of enhanced implementation on the effectiveness of the toolkit, the segmented regression model developed will be extended to include a covariate for the study implementation arm (control arm = 0; intervention arm = 1). We will explore adding control variables into our models if significant imbalances in NH characteristics (e.g., size, staffing ratios) are identified in our preliminary analyses. Based on data previously collected by our group [6], the expected rate of UTI treatment is ~4.0 per 1,000 resident-days. Assuming implementation of the UTI Toolkit leads to at least a 15% reduction in the UTI treatment rate and the facility dropout rate is <25%, our study will have 90% power to detect a statistically significant difference of this magnitude.

F. Program Evaluation

Please note that the evaluation methods that follow are currently still being developed and the content of this section will likely change. This section will be finalized and IRB protocols will be updated before commencing any the data collection described below.

F.1. Overview

A mixed methods evaluation structured around the RE-AIM framework (Reach, Effectiveness, Adoption, Implementation and Maintenance) [32] will be employed to compare the impact of the two implementation strategies using data submitted by facilities, surveys, interviews and observations. Statistical comparisons using generalized linear mixed models [33] will be employed to examine if differences in antibiotic utilization for treatment of UTI exist between NHs in the two study arms. Data from surveys, interviews and observations will be analyzed using a mixed-methods approach to ascertain the extent to which recommended toolkit practices have been implemented in study NHs.

F.2. Subjects

F.2.1. Nursing Home Residents

As the intervention in this study is targeted at the organizational level and not the individual resident, we will not be recruiting residents as part of this study. In order to assess the impact of the UTI Improvement Toolkit on antibiotic prescribing, research staff will review the aggregate data about residents who have been treated for a UTI before and after the intervention to determine if documentation reflects a change in management practices in the facility. The resident data that will be collected are listed in the table in section E.1, under “Resident Level Outcomes”.

F.2.2. Nursing Staff and Providers

We will be recruiting nursing staff and providers in order to evaluate facility engagement in the implementation of the toolkit and the factors that influence implementation.

F.3. Subject Recruitment

Within each nursing home, the Practice Manager will be contacted to identify staff to participate in interviews. These individuals will be sent an email (developed by the research team but sent by their practice manager) that invites them to participate and lists a telephone number to call the research team about questions or concerns. An information flyer (i.e., recruitment flyer) will also be attached to the email. If any of the clinician staff do not use an individual email account for work, the email text will be sent as a letter to those potential participants by postal mail, with a hard copy of the information flyer enclosed.

For the interviews and observations, all facility-employed and per diem nursing staff (certified nursing assistants [CNA], licensed practical nurses [LPN] and registered nurses [RN]) who are responsible for the care of >3 residents will be included in the study. For the questionnaires, all nursing home clinical care staff will be invited to participate.

F.4. Data Collection Procedures We will collect data on each part of the RE-AIM framework: Reach, Effectiveness, Adoption, Implementation and Maintenance.

F.4.1. Reach

By means of interviews and surveys, we will collect data on the number of NHs that adopt the UTI Toolkit with fidelity. The surveys and the interview guides will be submitted for IRB approval via a change in protocol when developed at the end of Year 1.

F.4.2. Effectiveness

Some of the data mentioned above will also be used to assess Effectiveness. Specifically, the data on Nursing Home Quality Indicators and on Resident Level Outcomes (see Table in Section III. E.1) will be used here.

F.4.3. Adoption

We will collect data on the knowledge, attitudes, and perceptions of NH staff about the UTI toolkit. We will also use some of the data mentioned above, namely the data on Organizational Culture (see Table in Section III. E.1).

F.4.4. Implementation

To assess implementation, we will use a UTI toolkit fidelity checklist. We will also collect data to identify barriers to, and facilitators, of implementation. To collect these data, in addition to interviews and surveys, we will use a coaching workbook, on which the clinical coach will track their interactions with NH staff. Characteristics of these interactions, including length of encounters, number of participants, notable facility developments (e.g., leadership turnover, survey visits), progress made, barriers encountered and suggested strategies for overcoming identified barriers, will be detailed in this workbook.

F.4.5. Maintenance

Maintenance will be assessed by evaluating if participating nursing homes were able to sustain over a 9 month period the Nursing Home Quality Indicators and the Resident Level Outcomes (see Table in Section

III. E.1). We will also utilize qualitative interviews with staff (guides will be submitted for IRB approval via a change in protocol when developed at the end of Year 1) to assess facilitators and barriers to sustainability of the intervention in the NHs.

F.5. Data Analyses

A mixed methods evaluation approach structured around the RE-AIM framework [32] will be employed in this aim. **Reach** is the percentage of NHs that adopt the Wisconsin UTI Improvement Toolkit with fidelity. To compare differences in toolkit **effectiveness** between the two implementation strategies, the segmented regression model that will be developed (see Section III.E.4) will be extended to include a covariate for the study implementation arm (control = 0; externally-facilitated = 1). **Adoption** will be assessed both quantitatively (differences between NHs in the two implementation arms will be assessed by survey items measuring organizational readiness for change, staff knowledge, attitudes and perceptions about the UTI toolkit) and qualitatively using a thematic approach [34] structured around System Engineering Initiative for Patient Safety (SEIPS) framework [35]. Differences in the extent of **implementation** in NHs randomized to the two approaches will be assessed by comparing responses to a UTI toolkit fidelity checklist and qualitatively through analyses of data collected during staff interviews that focus on the identification of implementation strategies and barriers. Short-term **maintenance** of the effectiveness outcomes will be assessed utilizing an approach developed by Drs. Ford and Robinson [36]. To assess the impact of coaching, we will utilize the comparative conceptual framework proposed by Bond and Seneque [37] which suggests that organizational coaching occurs across one of five domains: managerial, consulting, mentoring, facilitating or coaching to classify the advice provided during the coaching interactions. Quantitative and qualitative data will be integrated in an attempt to explain implementation success and failures using a formal triangulation approach [38]. Coaching notes, interview transcripts with NH champions, and notes generated during hosted peer-to-peer learning sessions will be analyzed thematically to identify common barriers NHs encountered and strategies employed to overcome them as well as the types of coaching instruction that were most impactful on facility implementation efforts.

F.6. Data and Record Keeping

F.6.1. Interviews

No identifiable information about the interview participants will intentionally be gathered. If any such information is mentioned during the interview, it will be removed from the audio file and transcript as soon as it is noticed by the research team. Interviews will be transcribed by a UW-Madison approved transcription service, Premium Business Services, which uses a HIPAA-compliant secure server to transfer files. Transcriptions will be reviewed and redacted for unintentional mention of personal health information or nursing home identifiers.

Audio files and transcripts will be stored on a secure password-protected server maintained by the Department of Medicine in the UW School of Medicine and Public Health. Only UW research team members will have access to these files through password-protected computers. Cleaned and de-identified transcripts may be stored on the local hard drives of the password-protected computers of UW research team members while the data are being analyzed.

F.6.2. Observations

The observations will be completed by clinical coach, who will not record any identifying information about the observation participant or any information about patients in their notes. Field notes will also be redacted for nursing home identifiers. Field notes will not contain PHI, as they relate to facility-level systems, which do not require patient-specific information.

The notes will be typed up by the clinical coach, carefully checked to remove any identifying information, and stored electronically. Each coach will retain a personal copy of their notes, which they will refer to in answering questions during data analysis. These files will be stored in a secure location convenient to the coach on a password-protected computer maintained by the Department of Medicine in the UW School of Medicine and Public Health. All clinical coaches will have access to their own notes, but only UW research team members will have access to the electronic notes of the entire research team, which will be stored on a secure password-protected server maintained by the Department of Medicine in the UW School of Medicine and Public Health. The coaches will destroy personal copies of their notes when the study is completed.

To protect identifiable subject information, we will store a list of contact information for clinic staff on a secure password protected server maintained by the Department of Medicine in the UW School of Medicine and Public Health. The list will only be accessible through password-protected computers of the UW researchers. The contact information will not contain any links to data and will be destroyed after the study is completed. All email correspondence with participants, both sent and received, will also be destroyed after the study is completed.

The audio files, de-identified transcripts and all other data will be destroyed after seven years, in compliance with UW's recommended retention period.

F.6.3. Surveys

As previously explained, we will use the UW Antibiotic Stewardship Program (ASP) survey, the UW organizational survey, the CHSRA/DHS Nursing Home Scorecard, and a survey to collect data on nursing homes that adopt the UTI Toolkit with fidelity and to identify barriers to, and facilitators of, implementation. We plan to either mail these surveys to the staff of the participating facilities or have them complete the surveys electronically through REDCap (ICTR instance). The surveys will be anonymous, and, for paper surveys, the participants will be asked to mail them to the research team by using a postage-paid return envelope that we will include with the surveys.

The completed surveys will be stored in a locked cabinet in the Department of Medicine in the UW School of Medicine and Public Health. Only UW research team members will have access to these documents on REDCap. The documents will be destroyed after seven years, in compliance with UW's recommended retention period.

Appendix: The Wisconsin Healthcare-Associated Infections in Long-Term Care UTI Improvement Toolkit

HAI UTI Toolkit Modules

1. Overview
2. How to Prevent UTIs?
3. When to test for UTIs?
4. When to treat UTIs?
5. How to treat UTIs?
6. Organizational tools

The WI HAI in LTC UTI Toolkit was built around the resident risk assessment and delayed action protocols developed by the PI of this project and another coalition member [11]. The conceptual framework of the UTI Toolkit focuses on three behavioral domains: (a) appropriate use of urine diagnostic tests in residents experiencing a change in condition; (b) appropriate treatment of a UTI; and (c) prevention of UTIs. The UTI toolkit is comprised of six modules (text box). The first module provides an overview of the toolkit and a rationale for antibiotic stewardship in NHs. The second through fifth modules cover the clinical decision pathways associated with the diagnosis and treatment of UTIs. The sixth module focuses on process and outcome tracking tools. A more detailed description of each module and its associated components are shown in the following table.

UTI Toolkit Module	Module Description and Overview
1. Overview	• Slide set and recorded audio presentation with rationale for antibiotic stewardship in nursing homes and introduction to the other modules of the WI HAI in LTC UTI Toolkit
2. How to prevent UTIs	• Appropriate use and management of indwelling urinary catheters<br>• Importance of and how to assess hydration status of the nursing home resident<br>• Importance of and how to collect a urine specimen from a resident with suspected UTI
3. When to test for UTIs?	• Role of testing in the over-treatment of UTI<br>• How to stratify residents into low and higher risk of UTI<br>• Risk-specific scripted language for nursing staff to employ in their interactions prescribers that promote active monitoring and delayed testing in low-risk residents<br>• Sample active monitoring care plan
4. When to treat UTIs?	• The accuracy of different clinical symptoms<br>• The different conditions that should be considered in residents with behavioral change<br>• Non-antibiotic therapies to consider in residents with isolated behavioral change<br>• Decision-support algorithm to help clinicians determine when it is appropriate to initiate antibiotic treatment UTI
5. How to treat UTIs?	• Effectiveness and risks of different antibiotics used to treat UTI<br>• Decision-support algorithm to help providers select the right antibiotic based on spectrum, renal function and residents allergy history<br>• Rationale for performing an antibiotic review<br>• Decision-support algorithm to help providers narrow antibiotic spectrum based on results of antibiotic susceptibility profile
6. Organizational Tools	• Urine culture tracking tool<br>• UTI treatment tracking tool<br>• Facility antibiogram development tool

Each of the toolkit components were developed through a rigorous review of the published theory and evidence as well as input from frontline clinicians who practice in Wisconsin NHs. While a small study performed in VA long-term care facilities has shown that a toolkit similar to the one developed by the WI HAI in LTC Coalition was effective in reducing rates of urine cultures and UTI treatment [46], the impact of this approach in community NHs remains unknown. VA long-term care facilities differ substantially from community NHs in many respects [47] and the types of barriers encountered in Wisconsin NHs that attempt to implement the UTI Toolkit and how these barriers can be effectively overcome are not known.

The process and outcome tracking tools included in the current version of the Wisconsin UTI Improvement Toolkit will be adapted and incorporated into a web-based tool that study NHs will use to track and visualize their process and outcome measures.

Cited Literature
