# Supplementary material for: Effect of a Standard vs Enhanced Implementation Strategy to Improve Antibiotic Prescribing in Nursing Homes: A Trial Protocol of the Improving Management of Urinary Tract Infections in Nursing Institutions Through Facilitated Implementation (IMUNIFI) Study
Source: JAMA Netw Open. 2019 Sep 11;2(9):e199526. doi: 10.1001/jamanetworkopen.2019.9526 (PMC6739723; doi:10.1001/jamanetworkopen.2019.9526)
Supplement: Supplement 2. — eTable. Project Timeline and Key Tasks [file jamanetwopen-2-e199526-s002.pdf]

Supplementary Online Content

Ford JH II, Vranas L, Coughlin D, et al. Effect of a standard vs enhanced implementation strategy to improve antibiotic prescribing in nursing homes: a trial protocol of the Improving Management of Urinary Tract Infections in Nursing Institutions Through Facilitated Implementation (IMUNIFI) study. *JAMA Netw Open*. 2019;2(9):e199526. doi:10.1001/jamanetworkopen.2019.9526

eTable. Project Timeline and Key Tasks

This supplementary material has been provided by the authors to give readers additional information about their work.

© 2019 Ford JH II et al. *JAMA Network Open.*

eTable. Project Timeline and Key Tasks

	2018									2019												2020												2021		
	1	2	3	4	5	6	7	8	9	10	11	12	13	14	15	16	17	18	19	20	21	22	23	24	25	26	27	28	29	30	31	32	33	34	35	36
	April	May	June	July	August	September	October	November	December	January	February	March	April	May	June	July	August	September	October	November	December	January	February	March	April	May	June	July	August	September	October	November	December	January	February	March
**Project Preparation Activities**																																				
IRB Approval																																				
Clinicaltrials.gov Registration																																				
Protocol Paper																																				
Confirm NH participation and recruit additional NHs as needed																																				
Develop HAI UTI Toolkit Kickoff Materials																																				
Develop Website																																				
Coach Preparation and Training																																				
**Project Launch Activities**																																				
NH Randomization																																				
Initial Data Collection																																				
Initial Kickoff Meeting																																				
**Project Implementation Activities**																																				
Control & Intervention NHs																																				
Only Intervention NHs (Externally facilitated)																																				
**Project Evaluation Activities**																																				
Follow up NH & QI Data Collection																																				
Adoption & Implementation Data Collection																																				
**Data Analysis & Dissemination of Findings**																																				
Statistical Analyses																																				
Dissemination of findings																																				

© 2019 Ford JH II et al. *JAMA Network Open.*
